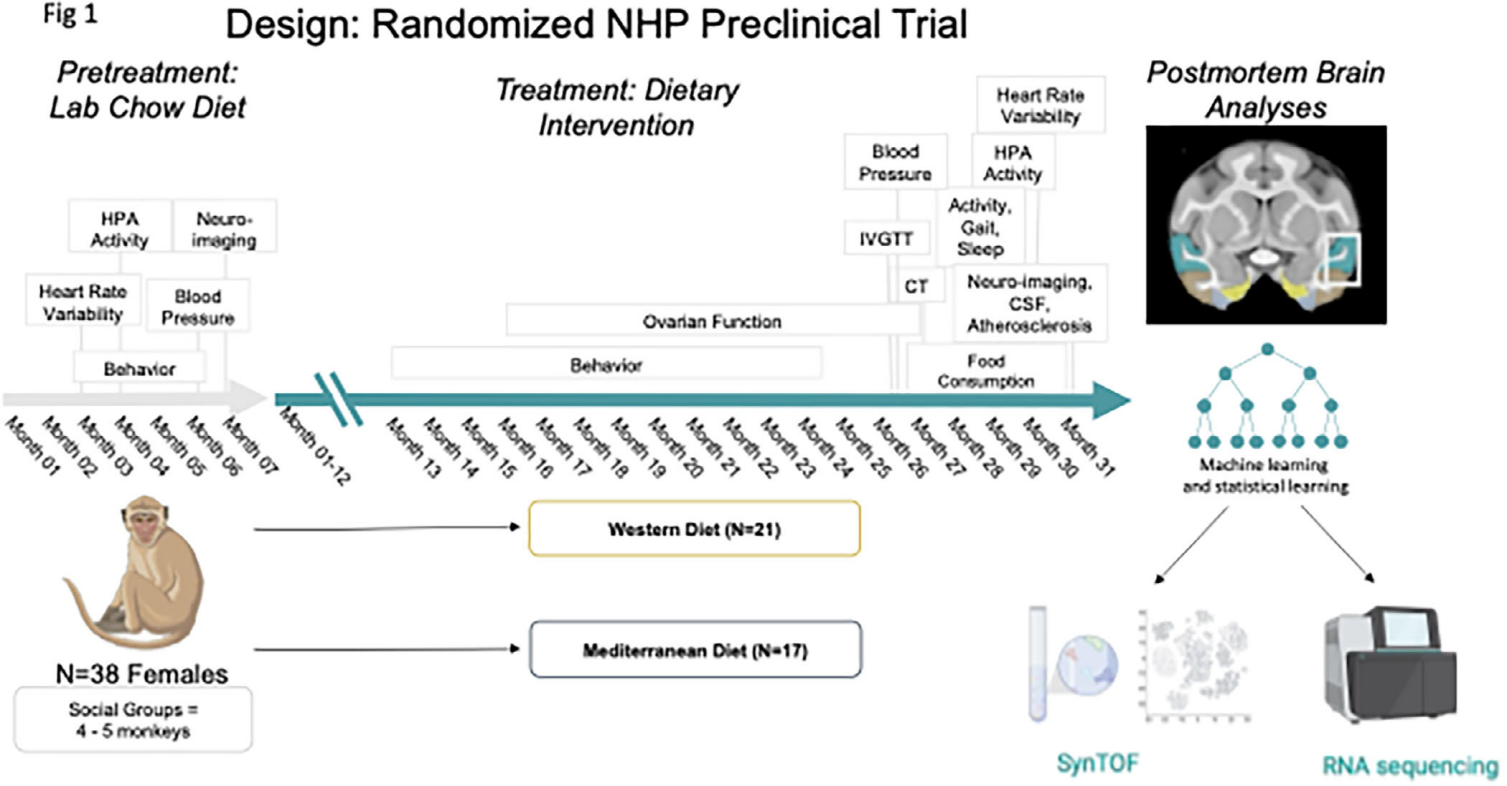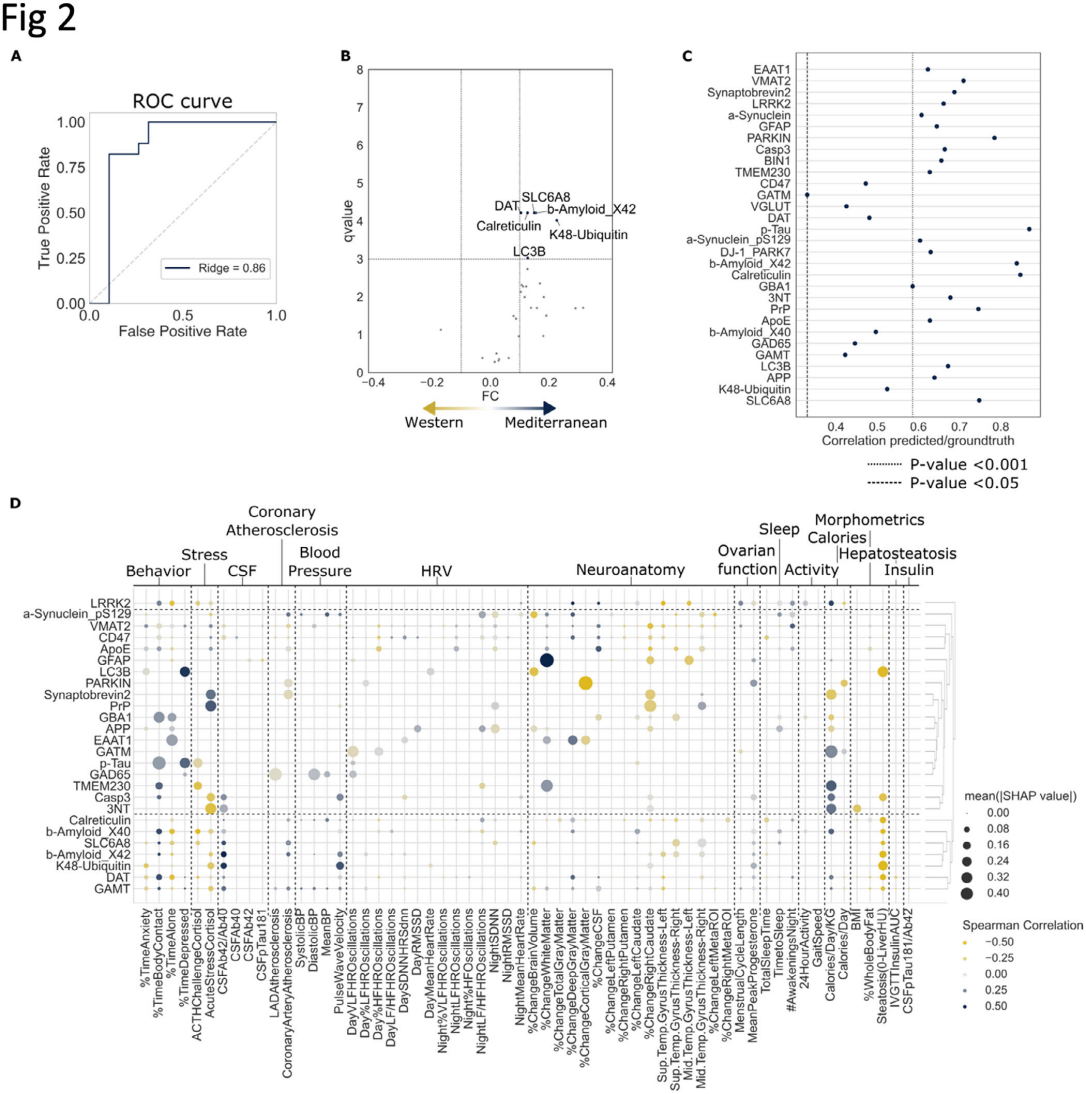# Effects of Mediterranean vs. Western Diets on the Cerebral Cortical Pre‐Synaptic Proteome in Nonhuman Primates

**DOI:** 10.1002/alz.089274

**Published:** 2025-01-03

**Authors:** Eloise Berson, Brett M. Frye, Amalia Perna, Thanaphong Phongpreecha, Sayane Shome, Geetha Clarke, Jacob D. Negrey, Nima Aghaeepour, Thomas J. Montine, Suzanne Craft, Thomas C. Register, Carol A. Shively

**Affiliations:** ^1^ Stanford, Palo Alto, CA USA; ^2^ Wake Forest University School of Medicine, Winston‐Salem, NC USA; ^3^ Stanford University, Stanford, CA USA; ^4^ Stanford University, Palo Alto, CA USA; ^5^ Department of Pathology, Stanford University School of Medicine, Stanford, CA USA; ^6^ Wake Forest Alzheimer’s Disease Research Center, Winston‐Salem, NC USA

## Abstract

**Background:**

Western and Mediterranean diets differentially affect cerebral cortical gene expression, brain structure, and socioemotional behavior in middle‐aged female nonhuman primates (NHP) (*Macaca fascicularis*). In this study, we investigate the effect of diet on brain molecular composition.

**Method:**

Using a machine learning approach, we quantified the impact of these diets on the presynaptic proteome in the lateral temporal cortex determined by synaptometry by time of flight (SynTOF) mass spectrometry and examined associations between the proteome, transcriptome, and an array of multisystem phenotypes. For this, we consider NHP fed with Mediterranean (n = 17) or Western (n = 19) diet for 31 months before brain retrieval (see study overview in **Figure 1)**.

**Result:**

Diet has a significant effect on presynaptic proteins (AUC = 0.86, Pvalue = 0.0002) (**Figure 2A**). We identified six presynaptic proteins (DAT, Aβ42, calreticulin, LC3B, K48‐Ubiquitin, SLC6A8) elevated in the presynaptic proteome bythe Mediterranean compared to the Western diet (p<0.05) (**Figure 2B**). Interestingly, we demonstrated that transcriptomic data from adjacent cortex predict all the SynTOF markers (pvalue <0.05) (**Figure 2C**). We found that the SPATA22 transcript was positively correlated with five SynTOF markers (LRRK2, TMEM230, GAMT and 3NT, Aβ40) (all p<0.05). Transcription Factor AP‐2 Gamma transcript (TFAP2C) was positively correlated with SynTOF markers pTau, CD47, and GAD65. Together, the multi‐system phenotypes significantly predicted 26 SynTOF markers, the strongest relationships were between GFAP and brain volumetrics (**Figure 2D**). Numerous SynTOF markers correlated with hepatosteatosis, suggesting the relationships between liver health and presynapses composition. SynTOF markers were also associated with behavioral and physiological measures of social‐environmental stress.

**Conclusion:**

Together these observations demonstrate that diet composition drives temporal presynaptic protein composition, that transcription profiles strongly predict the presynaptic proteomic profile, and that presynaptic proteins are closely associated with peripheral metabolism, stress responsivity, and socioemotional behavior. These data demonstrate the impact of diet composition on brain molecular composition.